# Evolution and virulence of porcine epidemic diarrhea virus following in vitro and in vivo propagation

**DOI:** 10.1038/s41598-024-62875-6

**Published:** 2024-05-29

**Authors:** Patumporn Jermsutjarit, Sunit Mebumroong, Parin Watcharavongtip, Hongyao Lin, Angkana Tantituvanont, Kampon Kaeoket, Pablo Piñeyro, Dachrit Nilubol

**Affiliations:** 1 Swine Viral Evolution and Vaccine Development Research Unit, Department of Veterinary Microbiology, Faculty of Veterinary Science, Chulalongkorn University, Henry Dunant Road, Pathumwan, Bangkok, 10330 Thailand; 2 MSD Animal Health Innovation Pte Ltd, Singapore, Singapore; 3 Department of Pharmaceutics and Industrial Pharmacy, Faculty of Pharmaceutical Sciences, Chulalongkorn University, Bangkok, Thailand; 4 Department of Clinical Sciences and Public Health, Faculty of Veterinary Science, Mahidol University, Nakhonpathom, Thailand; 5 Department of Veterinary Diagnostic and Production Animal Medicine, College of Veterinary Medicine, Iowa State University, Ames, IA USA

**Keywords:** Evolution, Genetics, Microbiology

## Abstract

Practice of inoculating porcine epidemic diarrhea virus (PEDV) in piglets generating feedback material might influence the genetic evolution and attenuation of PEDV. The study was conducted to evaluate evolutionary rate and attenuation following serial in vitro and in vivo propagation. In the study, PED-JPFP0-PJ, Passage 0 (P0), was isolated from infected pigs and serially passaged in Vero cells for 5 consecutive times, P1-P5. P0, P2 and P5 were then subjected to orally inoculate 3-day-old piglets. At 24 h post inoculation, intestines of each passage (F1), were collected, and subsequently sub-passaged in piglets for 2 additional passages (F2-F3). Virus titration, PEDV genomic copies number, VH:CD ratios, and immunohistochemistry were evaluated. S and ORF3 genes were characterized. The results of the study demonstrated that virus titer and virulence were negatively correlated with increased passages, both in vitro and in vivo. Increased substitution rate was observed in higher passages. The evolutionary rate of S gene was higher than that of ORF3. Seven aa changes at positions 223, 291, 317, 607, 694, 1114 and 1199, with reduced N-linked glycan were observed in P5F3. In conclusion, serial passage of PEDV, both in vitro and in vivo, influence the genetic development and the attenuation of PEDV.

## Introduction

Porcine epidemic diarrhea virus (PEDV) is an enteric pathogen causing disease in pigs characterized by vomiting, acute watery diarrhea, and high piglet mortality. PEDV is an envelope, positive-sense, single-stranded RNA virus belonged to order *Nidovirales*, family *Coronaviridae*, genus *Alphacoronavirus*. PEDV genome is approximately 28 kb in length encoding seven open reading frames; ORF1a, ORF1b, spike (S), nucleocapsid (N), membrane (M), envelop (E) and accessory protein ORF3 gene^1^.

S gene encodes S protein-a type I membrane glycoprotein containing 2 subunits; S1 and S2 domains. S1 domain is located outside the virion and binds to host specific receptors. It contains 2 receptor-binding domains (RBDs), residues of amino acid (aa) position 19-252 is N-terminal domain (S1-NTD) and aa at position 509-638 is C-terminal domain (S1-CTD)^2,3^. Moreover, S1 subunit is consisted of four domains; S1^0^, S1^A^, S1^B^ and S1^CD^^4^. S2 domain is truncated inside the virion and is responsible for membrane fusion by cleavage at S1-S2 border then promoting S2ʹ cleavage and entry into the host cytoplasm^5^. ORF3 gene is an accessory gene located between S and E genes. ORF3 functions primarily as an ion channel for permitting in viral replication and releasing new virions from infected cells^6^. It is speculated that ORF3 might be associated with virulence of the virus since serial passage of PEDV in vitro resulted in the deletion of 51 nucleotides (nt) in which is similar to two attenuated strains, CV777 and DR13^7^.

Presently, two PEDV genotypes including G1 and G2 are recognized^8^. The S gene is a distinguishing feature. G2 contains two insertions of 4 (^56^GENQ^59^) and 1 (^140^N) aa at positions 55-60 and 140, respectively, and a deletion of 2 aa (^160^DG^161^) at positions 160-161^9^. G2 has been reported as an emerging variant worldwide, and further evolves into subgroups G2a and G2b^10^.

Since its first emergence in 2007, PEDV has caused outbreaks throughout Thailand. The isolates circulating in Thailand are mainly variants in genotype G2 closely related to isolates in China genotype G2^11^. In late 2013, PEDV outbreaks with milder clinical symptoms were reported in several herds in Thailand. An epidemiological investigation reported that repeated outbreaks were caused by in the introduction of genotype G1^12^. G1 Thai isolates were genetically similar to that of modified live vaccines (MLV)^12^. At present, PEDV in Thailand is genetically diverse of which six subgroups in both genotypes G1 and G2 were previously reported^13^. The high genetically diverse was reportedly due to two mechanisms including substitution due to evolution and recombination with exotic strains^13^.

The common practice of oral feedback could also potentially accelerate virus evolution rate. This is a process where minced intestines of PEDV infected piglets are used to orally expose sows to field strains of PEDV. This is done either to all sows in a mass immunization protocol or to sows at pre-parturition to increase lactogenic immunity^14^. During an acute outbreak, oral feedback is first administered to all sows in the herd. Subsequently, swine practitioners attempt to prevent break-through outbreaks by administering oral feedback to sows at pre-parturition to increase lactogenic immunity for piglets. A common problem in endemic herds after an acute outbreak is a falloff in new outbreaks and hence a lack of infectious materials to conduct the feedback. Although pigs at all ages are susceptible to PEDV infection, pigs at lower than a week of age are the most susceptible with mortality approaching 100% and clear pathological lesions at intestine. The administration of intestines and feces from PEDV affected sows is not possible due to major issues on bacterial contamination and sows in PEDV endemically infected herds are less likely to develop clinical diseases. Therefore, producers commonly solve this problem by collecting and freezing intestine of PEDV affected piglets. The frozen intestine is used to regenerate more infectious materials using a biological seeder method. However, the supply of the frozen intestine is reduced when this managerial practice has been routinely implemented. Frozen intestines are then subjected for oral inoculation into 1-day-old colostrum deprived piglets. Intestines from these seeder piglets are then used as feedback materials. Producers may subsequently use infectious materials from these seeder animals to inoculate more seeder animals, to sustain stocks of infectious materials. Indirectly, this creates an in vivo passage model in the field and potentially increases genetic diversity of PEDV.

Anecdotally, field observations reported that serially passaged infectious material demonstrates reduced virulence and clinical diseases when inoculated into neonatal piglets using infectious material that has been serially passaged for more than twice. Our speculation is that this common practice may be accelerating the genetic diversity of PEDV due to multiple artificial passages in piglets, a practice that would normally take several field infection cycles to establish, resulting in reduced virulence due to changes in aa. We hypothesize that repeated passages in naïve piglets accelerate mutation in S and ORF3 genes, both of which are associated with PEDV replication. A key mechanism of evolution in RNA viruses is aa changes^15^. Therefore, a mimic feedback model was utilized to conduct serial passage in vivo. The objectives of the study were to examine the genetic evolution of S and ORF3 genes of PEDV through in vitro and in vivo serial passage, and to elucidate the associated alterations occurring at the genetic, aa, and secondary structure levels. Additionally, the study also explored the relationship of the genetic alterations with infectivity and virulence.

## Results

### Serial passage in vitro and in vivo decreased virus titers

**Figure 1:**
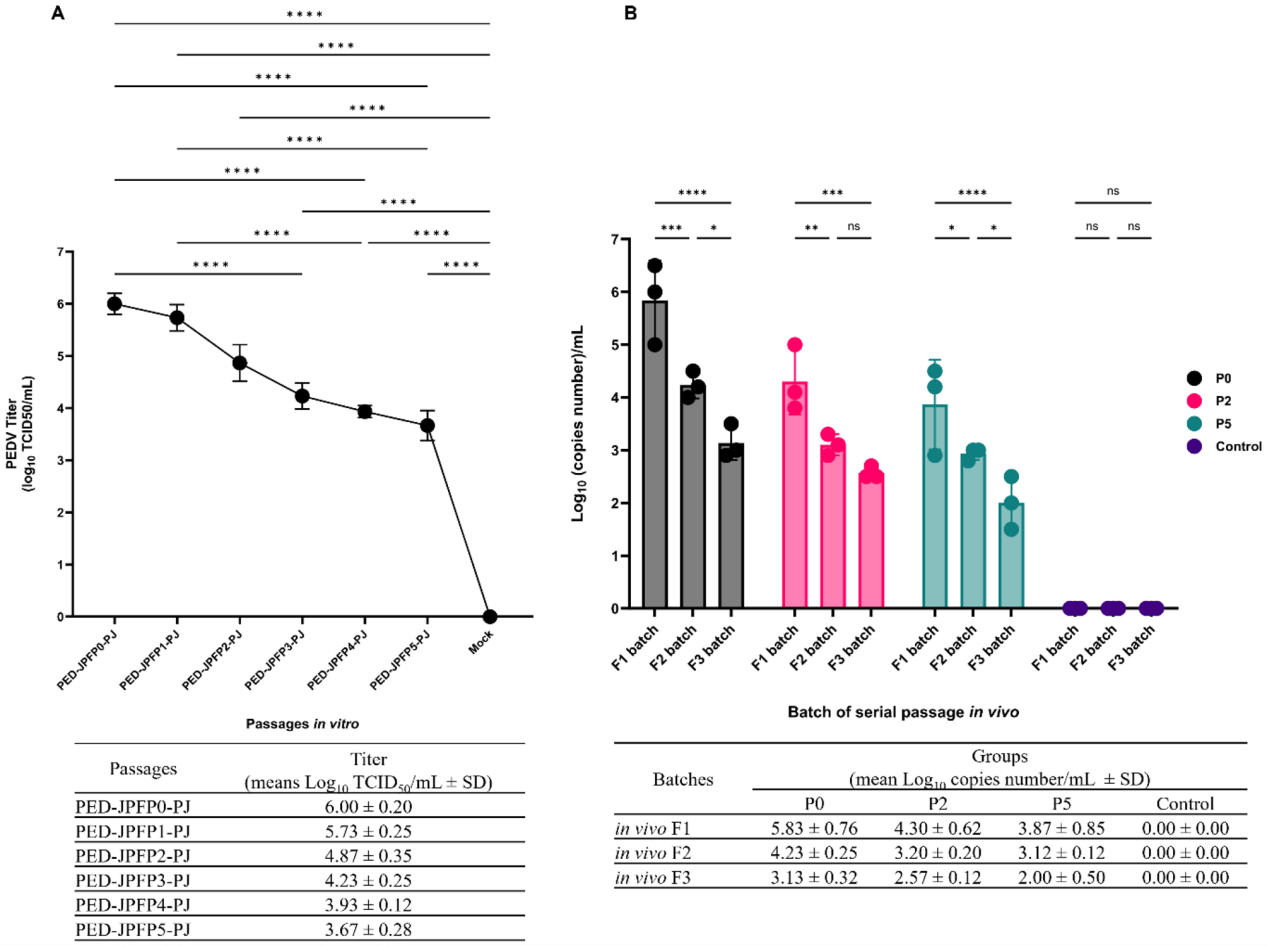
Comparison of PEDV titer between parental isolate, PED-JPFP0-PJ, and serially passaged 1st to 5th passage in vitro (**A**). Comparison of PEDV nucleic copy numbers between batches of serially passaged PED-JPFP0-PJ, PED-JPFP2-PJ and PED-JPFP5-PJ in vivo (**B**). Black bars represented the PED-JPFP0-PJ passage in piglets. Pink bars represented the PED-JPFP2-PJ passage in piglets. Green bars represented the PED-JPFP5-PJ passage in piglets. Dark purple bars represented the piglets in control group. The data showed as mean ± standard deviation (SD). ****P ≤ 0.0001, ***P ≤ 0.001, **P ≤ 0.01 and *P ≤ 0.1.

Virus titration was performed in all passages in vitro. Virus titers were compared to that of the parent isolate, PED-JPFP0-PJ. The results demonstrated that the parental strain PED-JPFP0-PJ had the significantly highest virus titer compared to its in vitro serially passaged variants. In contrast, PED-JPFP5-PJ, the 5th passage, had the lowest virus titer. Compared to the 3rd (PED-JPFP3-PJ), the 4th (PED-JPFP4-PJ) and the 5th (PED-JPFP5-PJ) passages, the virus titer of PED-JPFP0-PJ was significantly higher. However, the virus titer of PED-JPFP0-PJ was not different from that of the 1st (PED-JPFP1-PJ) and the 2nd (PED-JPFP2-PJ) passages (Fig. 1A). The results demonstrated that virus titer was reduced following in vitro serially passaged in Vero cells.

Small intestines of piglets inoculated with the PED-JPFP0-PJ, and the 2nd and 5th in vitro passage, together with in vivo serially passaged variants including P0F1-P0F3, P2F1-P2F3, and P5F1-P5F3, were subjected to virus quantification using qPCR (Fig. 1B). The results demonstrated that the small intestine from PED-JPFP0-PJ inoculated-piglets, P0F1, had the significantly highest PEDV genomic copy numbers compared to P0F2 and P0F3. P0F3 had the significantly lowest genomic copies.

A reduction in PEDV genomic copy numbers following in vivo serial passage of the original passage was also observed in the 2nd passage, PED-JPFP2-PJ. P2F1 had the highest genomic copy numbers compared to P2F2 and P2F3. P2F3 had the lowest PEDV genomic copy numbers. The PEDV genomic quantities of all isolates retrieved from the PED-JPFP2-PJ were consistently lower than that of all isolates retrieved from the parent isolate.

Similar to the genomic PEDV quantity observed in piglets inoculated with PED-JPFP0-PJ, genomic copy numbers of piglets challenged with PED-JPFP5-PJ, P5F1, had significantly highest genomic copy numbers compared to P5F2 and P5F3. P5F3 had the significantly lowest genomic copies.

It is speculating that virus titer reduced following in vitro passage in Vero cells is due to the unnatural host. The copy number increased following the in vivo passage in the natural host of the virus. However, the in vivo serially passaged, although in the natural host, resulted in the reduction of virus titer.

### Macroscopic intestinal lesions are most severe in low passage for both in vivo and in vitro passages

**Figure 2:**
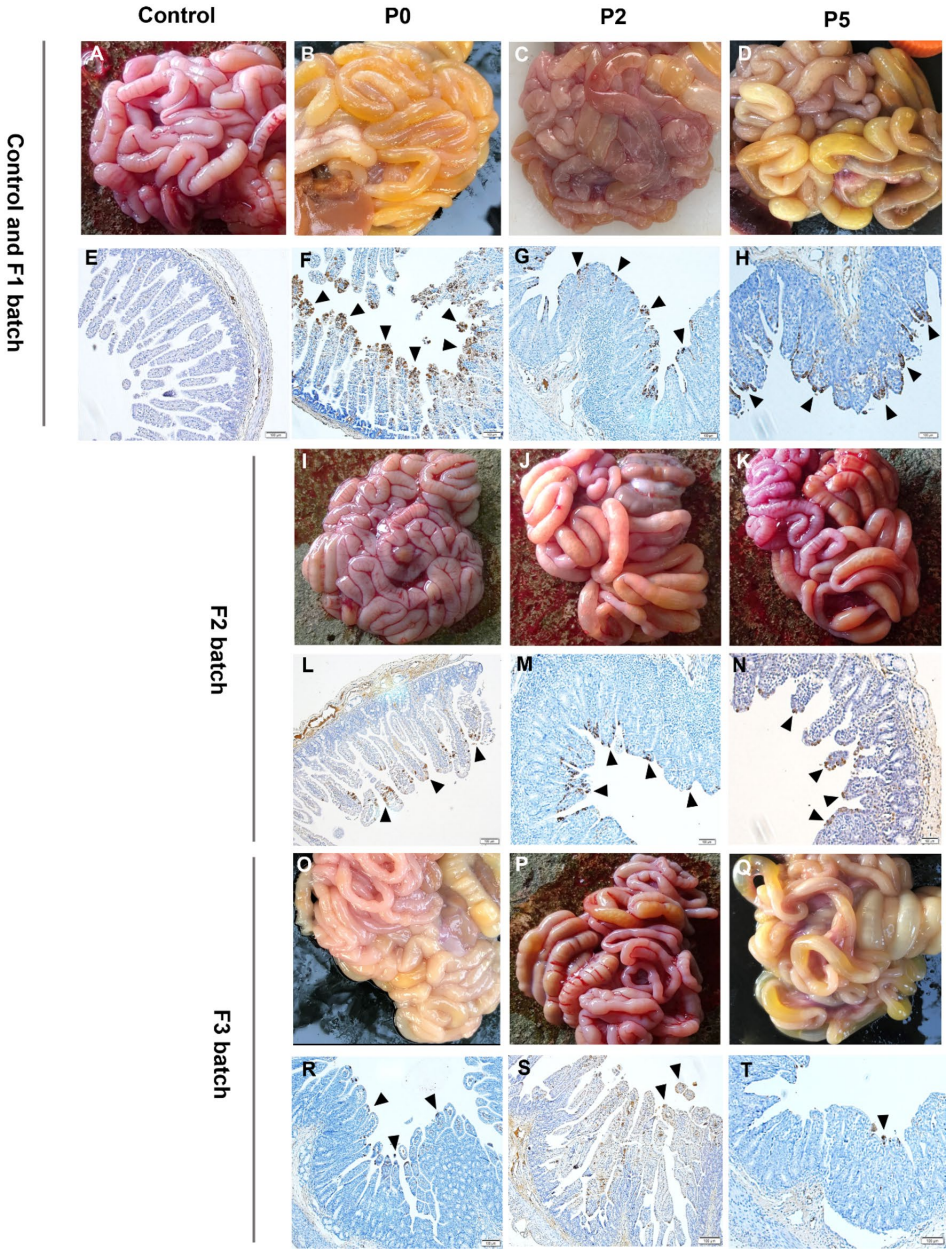
Histopathological and immunohistochemistry (IHC) analyses of small intestines. Representative intestines of each serial passaged-PEDV piglets’ batch were shown in figure. Macroscopic lesion, including control group (**A**), P0F1 (**B**), P0F2 (**I**), P0F3 (**O**), P2F1 (**C**), P2F2 (**J**), P2F3 (**P**), P5F1 (**D**), P5F2 (**K**), P5F3 (**Q**). IHC stained distal jejunum tissue sections of different groups are as follows; control group (**E**), P0F1 (**F**), P0F2 (**L**), P0F3 (**R**), P2F1 (**G**), P2F2 (**M**), P2F3 (**S**) and P5F1 (**H**), P5F2 (**N**), P5F3 (**T**), respectively. PEDV nucleocapsid (**N**) proteins were detected only in cytoplasm of villi enterocyte as showed in brown color. Black arrows are indicated positive signal of IHC assay in the intestinal tissue. Original magnification × 100.

Macroscopic examination of piglets inoculated with PED-JPFP0-PJ, and its in vitro serially passaged isolates, PED-JPFP2-PJ, and PED-JPFP5-PJ are shown in Fig. 2. The results demonstrated that all PEDV-inoculated intestines displayed typical macroscopic lesions associated with PEDV infection characterized by thin and transparent small intestinal walls, compared to normal macroscopic lesions in negative control piglets (Fig. 2A). However, the severity of macroscopic lesion is different between isolates. The results demonstrated that macroscopic lesions were the severest in small intestines collected from piglets challenged with the parent isolate, compared to that of challenges with the PED-JPFP2-PJ, and PED-JPFP5-PJ. Small intestines collected from piglets challenged with PED-JPFP5-PJ were less severe compared to that of both passages.

Small intestines, P0F1, collected from piglets inoculated the parent isolate were thinnest in all 5 parts (Fig. 2B), followed by P0F2 (Fig. 2I) and P0F3 (Fig. 2O). Compared to PED-JPFP0-PJ, the macroscopic lesions of variant PED-JPFP2-PJ were less severe. The intestinal wall was thicker and less transparent, although yellowish fluid was contained in the lumen. Less severity of macroscopic lesions following in vivo serial passage was evidenced in the 2nd and 5th passage. Piglets inoculated with PED-JPFP2-PJ had the thinnest transparent wall and yellowish content fluid in all parts of intestinal lumen, especially in P2F1 (Fig. 2C), followed by P2F2 (Fig. 2J) and P2F3 (Fig. 2P). The piglets in the PED-JPFP5-PJ inoculated group displayed the least severity of the macroscopic lesions compared to that of PED-JPFP0-PJ, and PED-JPFP2-PJ inoculated groups. The intestinal wall was thicker, and yellowish fluid was only present in distal jejunum of small intestine. Inoculated piglets with PED-JPFP5-PJ had the thinnest transparent wall and yellowish content fluid in intestinal lumen, especially in P5F1 (Fig. 2D), followed by P5F2 (Fig. 2K) and P5F3 (Fig. 2Q). These findings suggested that increased passages in vitro and in vivo, in its the natural host, decreased PEDV virulence as demonstrated by less severity of macroscopic lesions.

### VH:CD ratios are lowest in low passage isolates for both in vitro and in vivo passages, especially in distal jejunum

**Figure 3:**
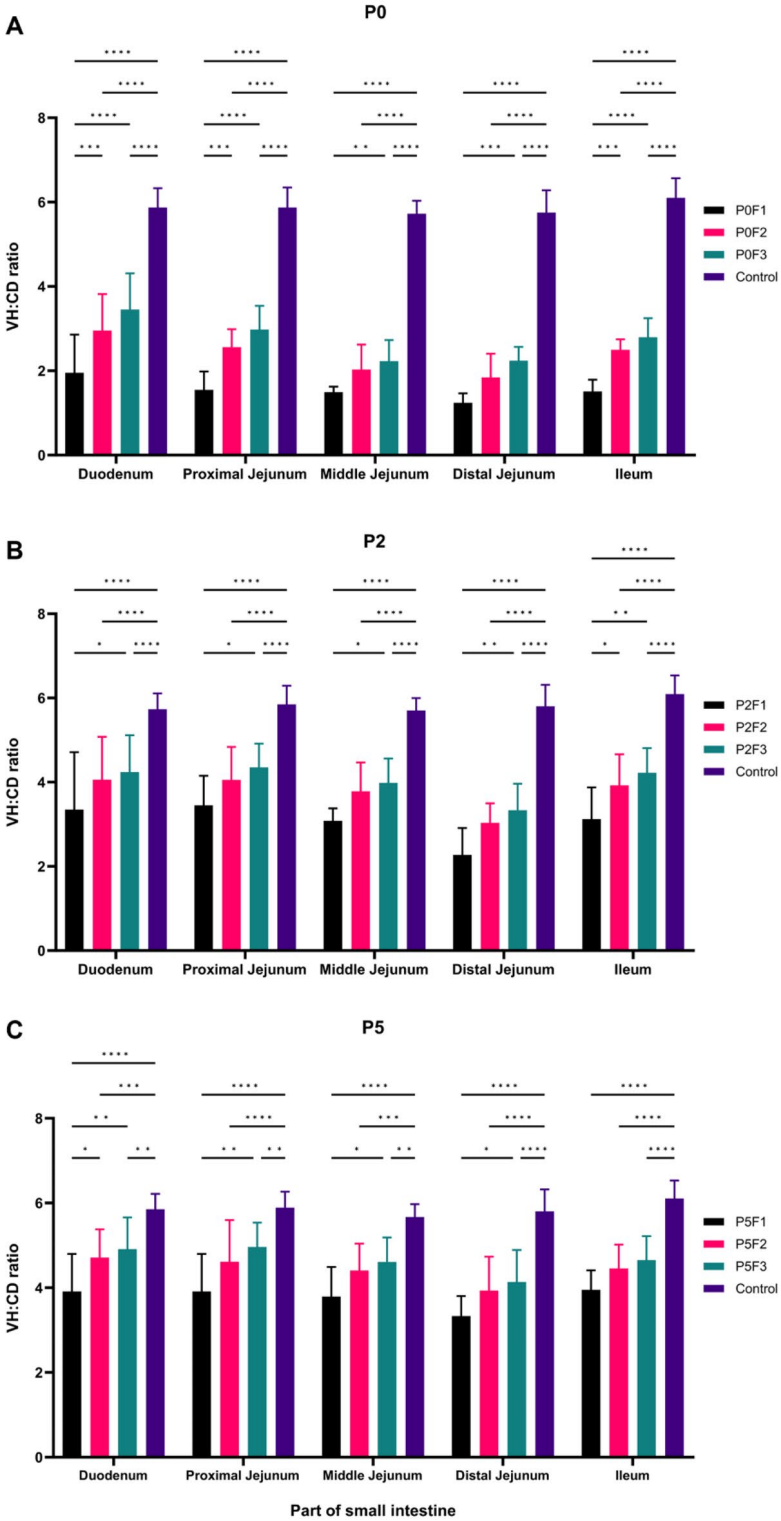
Villous height and crypt depth (VH:CD) ratios in duodenum, proximal jejunum, middle jejunum, distal jejunum, and ileum. VH:CD ratio of piglets challenged with the parent isolate, PED-JPFP0-PJ (**A**), the 2nd passage, PED-JPFP2-PJ (**B**) and the 5th passage, PED-JPFP5-PJ (**C**). Black, pink, green and purple bars represent different batches of serially passaged in vivo. The VH:CD ratios among the parts were statistically analyzed. Bars represented mean ± standard deviation (SD). ****P ≤ 0.0001, ***P ≤ 0.001, **P ≤ 0.01 and *P ≤ 0.1.

To further evaluate virulence of serially in vitro and in vivo passaged PEDV, VH:CD ratios were measured and the results are shown in Fig. 3. All PEDV-inoculated intestines displayed shortened VH:CD ratios, but the ratios were different between passages. It is suggested to the difference severity between passages.

Piglets inoculated with PED-JPFP0-PJ, had the significantly lowest VH:CD ratio, followed by P0F1, P0F2 and P0F3 (Fig. 3A). Piglets challenged with the PED-JPFP2-PJ had higher VH:CD ratio compared to small intestine from piglets challenged with PED-JPFP0-PJ (Fig. 3B). VH:CD ratio of P2F1 was lowest significantly different with P2F2 and P2F3. The similar pattern of higher VH:CD ratio was also observed in pigs challenged with PED-JPFP5-PJ. Piglets inoculated with PED-JPFP5-PJ had the significantly lowest VH:CD ratio, followed by P5F1, P5F2 and P5F3 (Fig. 3C). It is noteworthy that between 5 parts of small intestine, VH:CD ratio of distal jejunum was the lowest followed by middle jejunum, ileum, proximal jejunum and duodenum.

The results of VH:CD ratios support the previous findings of macroscopic lesions. Small intestines collected from piglets challenged with PED-JPFP0-PJ were the most severe compared to those challenged with PED-JPFP2-PJ and PED-JPFP5-PJ. Small intestines collected from piglets challenged with PED-JPFP5-PJ were less severe compared to that of both passages. These findings suggested that the in vivo serial passage resulted in decreasing PEDV virulence as evidenced by higher VH:CD ratios. Interestingly, decreased VH:CD ratios were mainly observed in middle to distal jejunum, indicating the target region of PEDV infection.

### IHC scores as demonstrated by PEDV N antigen significantly lower in higher passages

**Figure 4:**
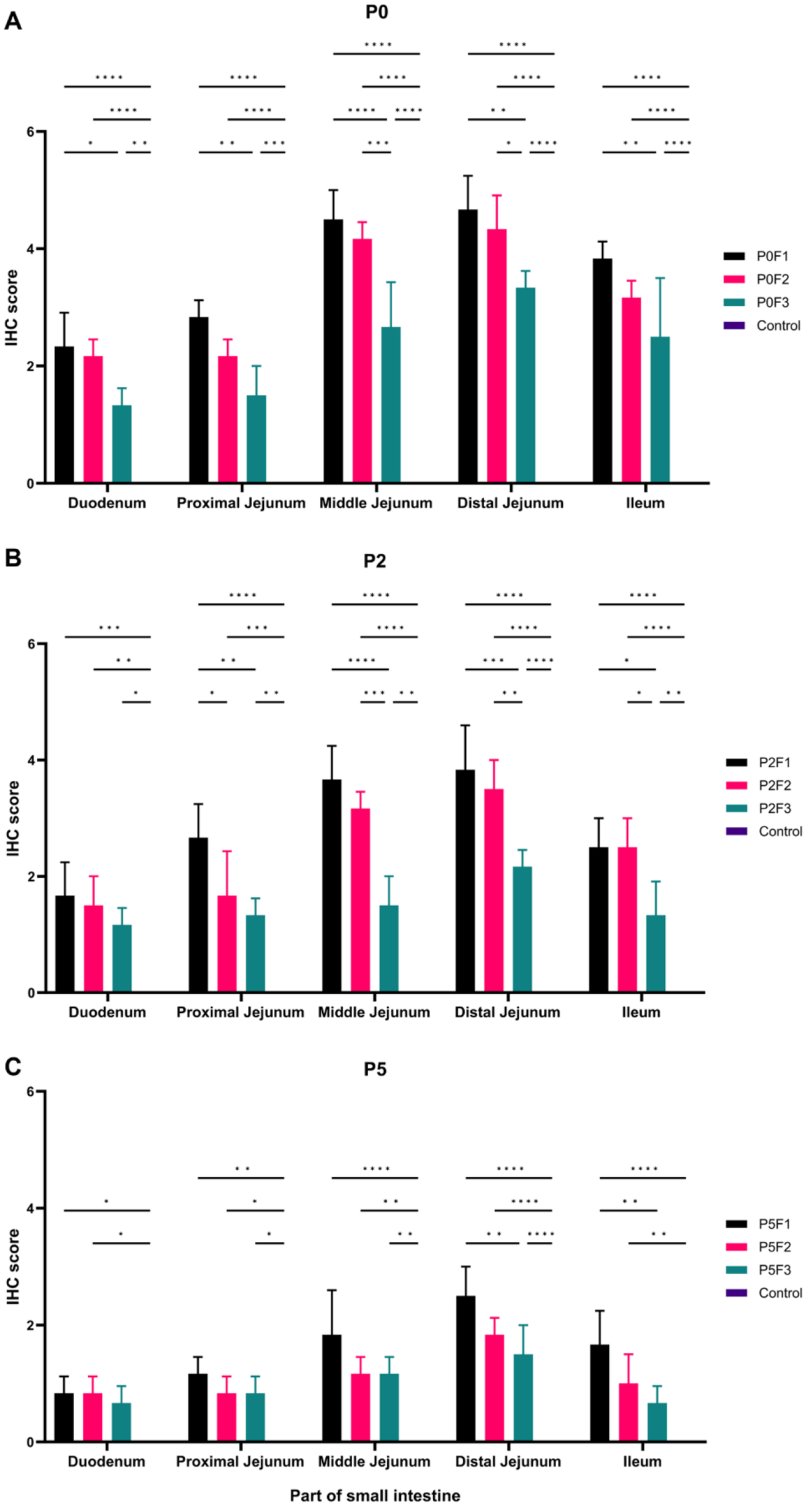
Immunohistochemistry (IHC) score of piglets challenged with the parent isolate, PED-JPFP0-PJ (**A**), the 2nd passage, PED-JPFP2-PJ (**B**) and the 5th passage, PED-JPFP5-PJ (**C**). Black, pink, green and purple bars represent different batches of serial passaged in vivo. The IHC scores among the parts were statistically analyzed. The data showed as mean ± standard deviation (SD). ****P ≤ 0.0001, ***P ≤ 0.001, **P ≤ 0.01 and *P ≤ 0.1.

IHC scores were significantly highest in distal jejunum from piglets challenged with PED-JPFP0-PJ compared to that of challenges with the PED-JPFP2-PJ and PED-JPFP5-PJ (Fig. 4). The highest IHC scores were displayed in small intestine from PED-JPFP0-PJ challenged piglets, P0F1, especially in distal jejunum followed by middle jejunum, ileum, proximal jejunum and duodenum after P0F2 and P0F3 (Fig. 4A). PEDV N antigens stained of P0F1, P0F2 and P0F3 were presented in Fig. 2F, L and R, respectively. Small intestine, P2F1, collected from PED-JPFP2-PJ challenged showed lower IHC scores compared to parent isolate after P2F2 and P2F3 (Fig. 4B). IHC scores of P2F3 had lowest significant difference compared to parent isolate, N antigens staining were exhibited in Fig. 2G, M and S. The lowest IHC scores were showed in small intestine from PED-JPFP5-PJ inoculated group compared to PED-JPFP0-PJ and PED-JPFP2-PJ (Fig. 4C). The IHC scores of P5F3 were significantly highest in distal jejunum followed by middle jejunum, ileum, proximal jejunum and duodenum. The N antigens stained of P5F1, P5F2 and P5F3 were showed in Fig. 2H, N and T, respectively.

It is noteworthy that the results of IHC score were resembling to that of VH:CD ratio, as PEDV N antigen was dominant in distal jejunum. The presence of PEDV N antigen decreased significantly in pigs challenged with higher passage. These findings suggest the target region of PEDV infection.

### Increased serial passage in vitro and in vivo resulting in decreased nt and aa similarities due to aa substitutions in S and ORF3 genes

**Figure 5:**
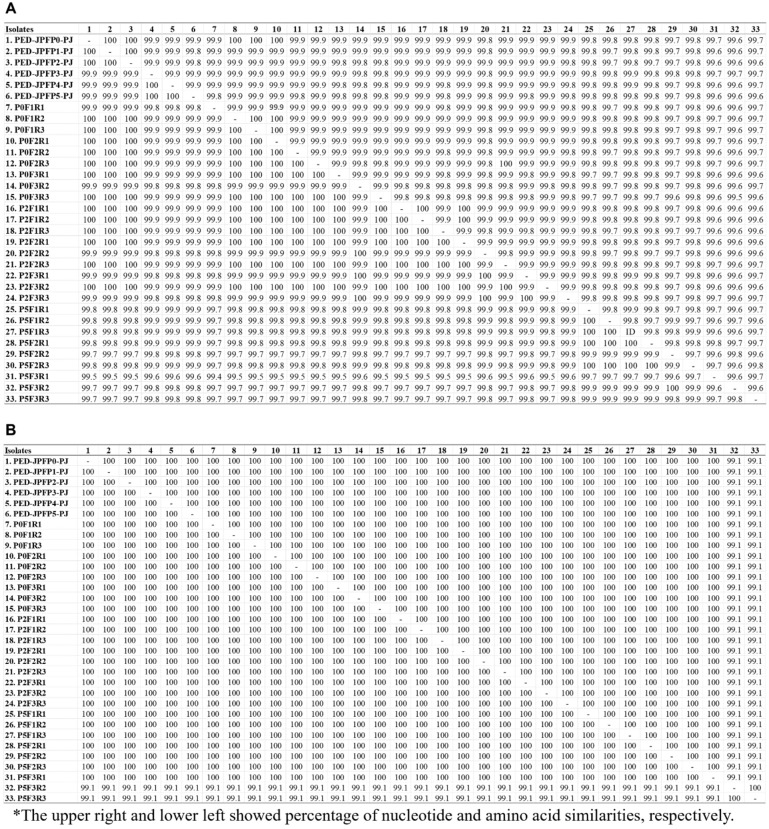
Comparison of the nucleotide and amino acid similarities (%) of parent isolate and its variants base on full-length S (**A**) and ORF3 (**B**) genes.

The complete S gene of PED-JPFP1-PJ and PED-JPFP2-PJ in vitro passages were genetically identical to the parent isolate with 100% similarity at nt and aa levels (Fig. 5A). In contrast, PED-JPFP3-PJ, PED-JPFP4-PJ and PED-JPFP5-PJ shared only 99.9% similarity at nt and aa levels. PED-JPFP3-PJ, PED-JPFP4-PJ and PED-JPFP5-PJ had 1 aa substitution at ^D^223^Y^ (Supplementary Fig. S2A).

PED-JPFP0-PJ, PED-JPFP2-PJ and PED-JPFP5-PJ were subjected for 3 additional in vivo passage in pigs. P0F2 was genetically identical to the parent isolate, while P0F1 and P0F3, with 99.9% and 99.9% identical to the parent isolate at nt and aa levels, had aa substitution at ^P^288^H^ and ^I^694^V^, respectively. P2F1 was genetically identical with PED-JPFP0-PJ and PED-JPFP2-PJ isolates. P2F2 and P2F3 were not genetically identical with parent and PED-JPFP2-PJ isolates. Amino acid substitution at ^I^694^V^ was found in 1 replicate of P2F2 and 2 replicates of P2F3. Two aa substitutions at ^D^223^Y^ and ^I^694^V^ were found in 3 replicates of P5F1. P5F2 showed the same aa substitutions as P5F1, but in 1 replicate, it was an additional aa substitution at ^L^1,114^I^. In P5F3, 7 aa substitutions were identified at ^D^223^Y^, ^L^291^H^, ^E^317^K^, ^P^607^L^, ^I^694^V^, ^L^1,114^I^ and ^T^1,199^A^.

In contrast to the complete S gene, based on the complete ORF3 gene, all 5 in vitro passages were genetically identical with 100% similarity at nt and aa levels (Fig. 5B). In addition, in vivo passages of PED-JPFP0-PJ and PED-JPFP2-PJ demonstrated a 100% similarity at both nt and aa levels compared to the parent isolate. In in vivo passages of PED-JPFP5-PJ, P5F1 and P5F2, were genetically identical with 100% similarity at nt and aa levels with the parent isolate. However, P5F3 exhibited drastically change in which its ORF3 genes demonstrate only 99.1% similarity, at both nt and aa levels compared to PED-JPFP0-PJ and PED-JPFP2-PJ. Two aa substitutions, at ^F^80^V^ and ^I^164^V^ were demonstrated (Supplementary Fig. S2B).

### Serial passage caused increase substitution rate in both of S and ORF3 genes compared to parental isolate

Serial in vitro PEDV passages, from P0 to P5, were evaluated for their substitution rate (Supplementary Table S2). Based on complete S and ORF3 genes, substitution rates of in vitro PEDV passages were lower compared to that of in vivo passages. Substitution rates were highest in serial in vivo passage, from P5F1 to P5F3 based on S and ORF3 genes. In summary, substitution rates in S and ORF3 genes were 4.16 × 10^–4^ (± 4.43 × 10^–7^; SEM) and 5.37 × 10^–4^ (± 1.85 × 10^–5^; SEM) for all serial passages, both in vitro and in vivo, from the original isolate, PED-JPFP0-PJ. S gene demonstrated a higher substitution rate compared to ORF3.

### Antigenicity and hydrophilicity changes accompanied amino acid changes in high passage isolates

**Figure 6:**
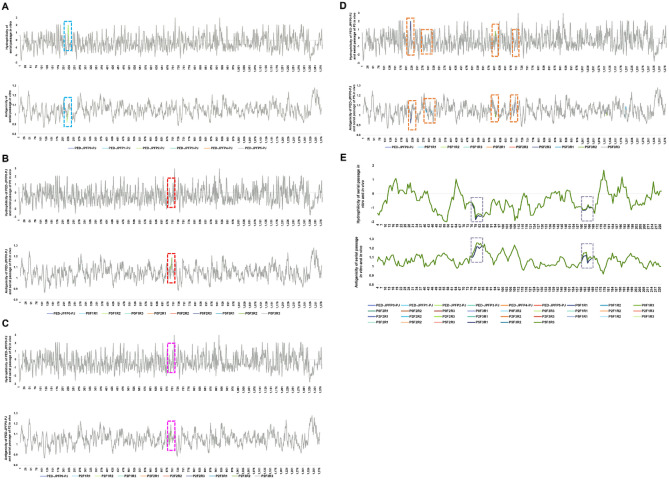
Hydrophilicity and antigenicity indices based on amino acid sequences of S and ORF3 proteins. Blue dashed boxes showed the different regions displaying variances between in vitro isolates (PED-JPFP0-PJ, PED-JPFP1-PJ, PED-JPFP2-PJ, PED-JPFP3-PJ, PED-JPFP4-PJ and PED-JPFP5-PJ) (**A**). Red dashed boxes showed the different regions displaying variances between PED-JPFP0-PJ and its variant in vivo (**B**). Pink dashed boxes showed the different regions displaying variances between PED-JPFP0-PJ and its variant, PED-JPFP2-PJ, in vivo (**C**). Orange dashed boxes showed the different regions displaying variances between the PED-JPFP0-PJ and variant of PED-JPFP5-PJ, in vivo (**D**). Difference regions rely on ORF3 gene, which is presented serially passaged in vitro and serially passaged in vivo as showed in gray dashed boxes (**E**). The deduced amino acid was predicted of hydrophilicity and antigenicity. Hopp-Woods hydrophilicity plot and Kolaskar & Tongaonkar antigenicity were created using Protscale Expasy (http://web.expasy.org/protscale) and IEDB Analysis Resource (http://tools.immuneepitope.org/main), respectively.

Antigenicity and hydrophilicity indices of complete S and ORF3 polyproteins between parent isolate, and in vitro passages, and in vivo passages were analyzed (Fig. 6).

Antigenic and hydrophilicity indices between the parent isolate, and its 1st and 2nd in vitro passages, were identical. The difference was observed in its 3rd, 4th, and 5th passages at aa220-223 in which was located in S1^A^ subdomain (Fig. 6A). In in vivo passages, P0F3 and P2F3 demonstrated different antigenic and hydrophilicity indices in S1^CD^ subdomain in which were located at aa690-694 (P0F3) (Fig. 6B) and aa688-696 (P2F3) (Fig. 6C), respectively. This is considered similar region. The 1st in vivo passage of PED-JPFP5-PJ was not different. However, four difference regions were observed between PED-JPFP0-PJ and P5F2 and P5F3. The four different regions were located in S1^A^ subdomain at aa220-223 and aa288-317, S1^B^ subdomain at aa604-608, and S1^CD^ subdomain at aa685-694 (Fig. 6D).

Based on ORF3 gene, antigenic and hydrophilicity indices demonstrated two different regions, between the parent isolate and the P5F3 at aa78-82 and aa161-166 (Fig. 6E).

### Amino acid substitutions predicted secondary structure changes in S protein

**Figure 7:**
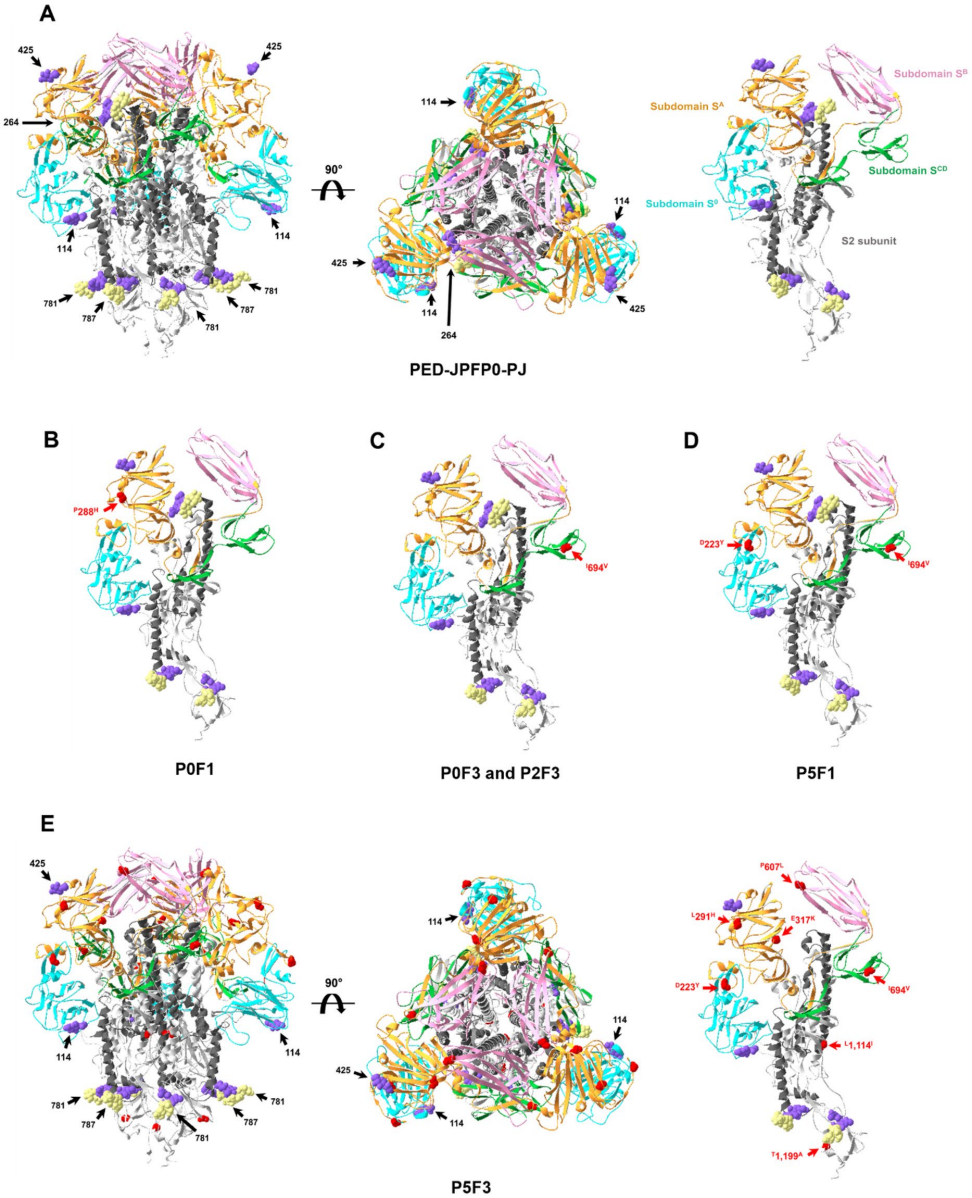
The S protein secondary structure of PED-JPFP0-PJ and its variants. The figures showed secondary structure of PED-JPFP0-PJ (**A**), P0F1 (**B**), P0F3 and P2F3 (**C**), P5F1 (**D**) and P5F3 (**E**), respectively. S1 subunit is represented in blue (S1^0^ subdomain), orange (S1^A^ subdomain), pink (S1^B^ subdomain) and green (S1^CD^ subdomain). S2 subunit is represented in grey color. Moreover, dark grey color is represented fusion protein of S2 subunit. N-linked glycans are showed in purple (without mannose) and pale yellow (mannose residues). Labels are represented position of carbohydrate linked asparagine residues. Amino acid substitutions are showed in red color.

Antigenic sites in S secondary structure were modelled based on the aa substitutions. The secondary structure displayed in Fig. 7, blue, orange, pink, green, and grey colors represented S1^0^, S1^A^, S1^B^ and S1^CD^ subdomains in S1 subunit, and S2 subunit, respectively. Amino acid substitutions were labeled in red color. The secondary structures of P0F1 and P0F3 were similar to that of the parent isolate, but 1 aa substitution of each at residues 288 and 694, respectively, were demonstrated (Fig. 7B and C). Interestingly, the secondary structure of P2F1 was similar to the parent isolate, however the secondary structure of P2F3 had 1 aa substitution at residue 694 in which is similar to the change of aa position in P0F3. Compared to P0F3 and P2F3, the secondary structure of P5F1 was similar to P0F3 and P2F3 with one more 1 aa substitution at residue 223 (Fig. 7D). The highest aa substitution was observed in P5F3. The secondary structure of P5F3 presented 7 aa substitutions at residue 223, 291, 317, 607, 694, 1,114 and 1,199 (Fig. 7E), compared to the parent isolate. Interestingly, aa substitutions in P5F3 were mainly located in S1 subunit in which functions as a binding domain and neutralizing epitope.

### The number of N-linked glycans decreased in P5F3

Twelve prominent N-linked glycans were identified on the S trimer surface of the parental strain, P0F1, P0F3, P2F1, P2F3 and P5F1, except P5F3 (Fig. 7). These 12 prominent N-linked glycans are located in S1^0^ and S1^A^ subdomains, and S2 subunit at aa positions 114, 264, 425, 781 and 787 of which are asparagine (N) residues. Four glycan sites at N114, located in S1^0^ subdomain, and N264, located between S1^A^ subdomain and S2 subunit, had 3 and 1 positions, respectively. S1^A^ subdomain contains additional glycan site at N425 that has 2 positions on the S trimer surface. Six glycan sites are predicted at positions N781 and N787 at the N-terminal of the S2 subunit, with 2 positions per monomer. N-linked glycan sites at 114 and 425, and at 264, 781 and 787 are without (purple color) and with mannose (pale yellow color) residues, respectively. In contrast, N-linked glycans reduced from 12 to 10 sites in P5F3, with N264 and N425 disappeared.

## Discussion

Theobald Smith’s work from the previous century suggests that pathogens will eventually evolve toward avirulence, possibly through serial passage through hosts^16^. From a One Health perspective, RNA coronaviruses continue to be the focus of both veterinary and medical scientists. The COVID‐19 pandemic has revived debate on if host–parasite interactions should evolve towards avirulence. Most common human coronaviruses have evolved toward causing little mortality or morbidity through generations of coexistence and in vivo passage through human hosts^17^. From a veterinary perspective, PEDV continues to be a critical RNA coronavirus affecting the swine industry. Our study thus serves as an accelerated model of continuous passage in vivo in an endemically infected population. The study accelerates the passage rate through an artificial passage which is incidentally similar to common field practice used in swine production. The practice is called feedback method. Through this model, understanding of changes resulted from repeated in vivo passage, and the potential future of PEDV as it evolves in the swine population are expected to be provided, and whether or not there is potential of PEDV to evolve toward avirulence or co-existence with host. This may also serve as a model for studies of future emerging coronaviruses in animals in which are often spillover hosts to humans.

Due to the abundance of genetic diversity and higher evolutionary rates of PEDV in Thailand compared to other regions^13^, it was speculated that serial in vivo passage of PEDV in pigs would influence the genetic development. The serial in vivo passage of PEDV is attenuated to mimic the field practice of feedback generation in biological seeder model of PEDV. The results of the present study provide strong evidence proving this speculation by demonstrating the reduction of macroscopic lesions and PEDV genomic copy numbers in higher passage in vivo. The present study includes notable aa changes in the S and ORF3 genes of the PEDV through serial passage, both in vitro and in vivo. The reduction in nt and aa similarities at higher passage levels, both in vitro and in vivo, leads to increased aa substitutions. Due to increased aa substitutions at higher passage, increased differences in antigenicity and hydrophilicity is observed between regions of complete S and ORF3 polyproteins in comparison to that of the parent isolate, PED-JPFP0-PJ. Increased differences in antigenicity and hydrophilicity result in structural changes on the surface of PEDV S protein, particularly in the receptor binding domain (RBD) and in the neutralizing epitopes (NE). The genetic changes in both RBD and NE affect the charge and polarity of the S protein, potentially reducing the virulence of the virus and its ability to propagate into their target host intestinal cells. Both pieces of evidence were supported by pigs displaying none to mild clinical diseases associated with PEDV infection following the challenge with higher passages of PEDV. This was in conjunction with a low virus titer detected in higher PEDV passage. Sows orally exposed to the high passage intestines of PEDV that are used as feedback, might not induce an immune response properly, or could potentially induce a protection that is not homologous to field PEDV. This results in an incomplete protection, consequently causing a PEDV outbreak in a herd. Based on our experiments, repeated immunization with feedback using higher passage of PEDV, both in vitro and in vivo, results in reduction of virulence of PEDV, which offers limited protection.

The S glycoprotein of PEDV, located on the surface outside of the viral envelope, plays a key role in binding to specific receptors, facilitating virus entry into host cells. It is structurally divided into S1 and S2 domains. The S1 domain comprises of four core subdomains including S1^0^, S1^A^, S1^B^ and S1^CD^. The S1^B^ contains the major receptor binding domain (RBD), located at aa510-640^18,19^. This domain binds to aminopeptidase N (APN). Subdomain S1^A^ and S1^CD^ contain neutralizing epitopes^20,21^, in which presently 5 regions including aa499-638, aa636-789, aa748-755, aa746-771, and aa1368-1374 have been reported^20,22,23^. In the present study, it was demonstrated that all PEDV passages, both in vitro and in vivo, have aa substitutions in 7 positions in P5F3. The P5F3, representing the highest passage in vivo, had a total of 5 major aa substitutions which are the highest aa substitution number observed in comparison to other lower passages. The 5 aa substitutions are located in regions including S1^A^, RBD and CO-26K equivalent epitope (COE), a core neutralizing epitope of coronaviruses. Amino acid substitutions in these regions were demonstrated on reduced binding affinity, antigenicity, pathogenicity and neutralizing activity of PEDV^24,25^. Two studies have been conducted previously to evaluate an association between virulence and the substitutions in RBD and COE. In one of the two previous study, PEDV CT strain was serially passaged in Vero cells for 120 passages^26^. The 10th (P10), 64th (P64) and 120th (P120) passages were subjected for oral inoculation in piglets. It was demonstrated that pigs inoculated with a high PEDV passage (P64 and P120) displayed milder clinical disease compared to pigs inoculated with lower passages (P10). P64 and P120 had 2 different aa substitutions in S1^A^ (^D^265^A^) and RBD (^F^635^R^) compared to P10, in S gene. In another study, PEDV strain FJzz1 was serially passaged in vitro for 200 passages^27^. Subsequently, the 20th (P20) and 200th (P200) passages were inoculated orally into piglets. Pigs inoculated with P200, with 3 aa changes (^T^502^I^, ^T^555^S^ and ^G^600^S^) in the COE epitope, displayed low pathogenicity with no obvious clinical signs, and no viral shedding, compared to high pathogenicity in pigs challenged with P20.

N-linked glycans are accessory carbohydrates hanging on the S trimer surface, which are important for appropriate folding and viral entry^28,29^. In the present study, 12 N-linked glycans were demonstrated in the parental strain, PED-JPFP0-PJ, as well as in P0F1, P0F3, P2F1, P2F3 and P5F1. Interestingly, in P5F3, the highest passage in vivo, only 10 N-linked glycans were found. Two glycan motifs at sites N264 and N425 in the S1^A^ subdomain in P5F3 disappeared. The N264 is a unique N-linked glycan of PEDV which is sandwiched between S1^A^ and S2 subunit, functioning to stabilize prefusion S conformation during virus infection^21^. We speculated that the loss of unique N264 glycan is due to the 2 aa mutations, ^L^291^H^ and ^E^317^K^, which are located in the same S1^A^ subdomain as ^N^264. This was proven by a previous study in which aa at position 264 was mutated from N (Asn264) to D (Asp264) by constructing a plasmid using specific primers for the ^N^264^D^ sequence. The mutated virus was then subsequently transfected into HEK293F cells. It was demonstrated that the ^N^264^D^ mutation resulted in a strong tenfold reduction of S protein expression. The loss of N-linked glycans at N264 in this study potentially reduced the stabilization of S protein resulting in low quantity of virus titer and reduced virulence of PEDV at high passage in vivo.

The ORF3 protein functions as an ion channel that facilitates intracellular transport of ions that consequently results in the stability of the host endosomal vesicles. The stability of these vesicles enhances the replication, assembly and release of the virus, thus contributing to the pathogenesis of PEDV^30^. In the present study, two aa mutations in the ORF3 gene, ^F^80^V^ and ^I^164^V^, were identified only P5F3, the highest passage in vivo. The reduction in virus titer, genomic copy numbers, and pathogenesis was also observed following the challenge of this high passage. These 2 aa changes, ^F^80^V^ and ^I^164^V^, might potentially result in inefficient ORF3 function, subsequently reducing virus reproduction. The results of the present study are in accordance with a previous report in which the plasmid ORF3_NP12_ strain was constructed by site-directed mutagenesis targeting the 3 aa changes including ^T^70^I^, ^F^81^L^, and ^M^167^S^^31^. The recovery of virus progeny was evaluated by co-transfecting each expression plasmid with the PEDV infectious clone into VeroE6-APN cells. It was demonstrated that the PEDV ORF3_NP12_ isolate contains ^F^81^L^ and ^M^167^S^ aa mutations. These 2 aa mutations correlate with the inhibitory activity of the ORF3 gene, subsequently impairing the rescue of PEDV in vitro, as evidenced by decreased levels of viral RNA and virus titers. Therefore, the 2 aa mutations, ^F^80^V^ and ^I^164^V^, in the present study closely corresponds to those in the previous study, suggesting that they inhibit the function of ORF3, resulting in decreased virus replication.

This study reports the substitution rate of S and ORF3 genes among passage was regularly in range. The substitution rate of PEDV S gene ranged from 1.67 × 10^–4^ to 1.04 × 10^–3^ s/n/c. It is noteworthy that lower passages, P1, had lower substitution rate (1.67 × 10^–4^) compared to a higher passage, P5. The accumulated substitution rate of Thailand's field strain PEDVs was 2.51 × 10^–3^ substitution per site per year (s/s/y)^13^, which is relatively close to the substitution rate of a high PEDV passage, P5. The highest substitution rate has been identified in P5F1 to P5F3 (1.04 × 10^–3^ s/n/c), with only 5 replications in vitro, and 3 replications in the host. The nucleotide substitution rate of these serial PEDV passages is similar to the wildtype, collected from the outbreaks from 8 years (2008–2015). The result supported that increased sub passages both in vitro and in vivo will increase viral evolution.

It is noteworthy that pigs orally inoculated with high passages of PEDV displayed milder clinical diseases compared to those exposed to low passages. Macroscopic lesions of the intestine were also milder, which were supported by higher VH:CD ratio. Regardless of the passage of PEDV, PEDV targets villous enterocytes. This correlated with N antigen and high VH:CD ratio observed in villous enterocytes of jejunum. Previous studies reported that the villous enterocytes are the cell tropism of PEDV^32,33^. The surface of villous enterocytes has been indicated to have dominant porcine aminopeptidase N (pAPN) acting as the cellular receptor for PEDV^34^. The S1 domain is majorly recognized by the pAPN receptor, facilitating entry into cells through membrane fusion and initiating the replication cycle. Mature virions are released through a cytolytic mechanism, resulting in CPEs in cell culture or pathogenesis in a natural host. Through the cytolytic release of virions, PEDV rapidly infects enterocytes, leading to apoptosis, which in turn causes villous atrophy^35^, as observed by VH:CD ratios. Present study found jejunum display numerous instances of villous atrophy in comparison to other anatomical regions, particularly in distal jejunum. The VH:CD ratios were supported by IHC scores, in which higher VH:CD ratio and lower PEDV N antigens in tissue were observed. Thus, the findings of the present study suggest that villous enterocytes, especially in the distal jejunum, are a target tissue for the PEDV strain used in this study.

Changes in aa in S and ORF3 genes may reduce the virus's ability to infect, thereby resulting in a decreased stimulation of the immune response against PEDV. Based on our experience, we observed that repeated immunization with feedback results in reduction of virulence of PEDV and offers limited protection. Typically, the virulent PEDV strain elicits a higher stimulation of protective immune response compared to that provided by the attenuated strain. Pigs inoculated with the virulent PEDV strain showed no clinical signs after challenge with the homologous virus. A comparison of the immune response stimulated by the virulent and attenuated variants of PEDV has been reported^36,37^. According to a previous study, the PEDV PC22A strain was serially passaged in Vero cells for up to 160 passages^38^. The P3, P100 and P120 passages were further studied for pathogenicity and immunogenicity^37^. Each pig inoculated with P3, P100, and P200 was orally challenged with a virulent P3 virus, and fecal viral shedding titers, as well as serum PEDV IgA, IgG, and VN antibody titers, were investigated. Both the P100 and P120 groups showed lower viral shedding and did not demonstrate differences in pathogenicity. In contrast, P3 group exhibited higher viral shedding compared to the P100 and P120 groups. In addition, the 3 variants differed significantly in immunogenicity. P3 induced the highest mucosal IgA levels compared to other groups, which represent a protective immune response against virus infection. On the other hand, comparison of immune responses between P100 and P120 revealed that P100 exhibited higher serum levels of PEDV IgA, IgG, and viral neutralization (VN) antibodies than P120. The higher passage may have been more attenuated, resulting in a less stimulated immune response. Considering the field situation, feedback utilizes the repeated passage of piglets' intestines as a material to stimulate a protective immune response in sows. The repeated passage in the natural host raises concerns about mutations that could potentially reduce the virulence of PEDV, thus diminishing the immunity generated. It is likely that sows inoculated with the homologous PEDV strain develop a higher level of mucosal immune response compared to those inoculated with attenuated PEDV. Our findings provide important insights into the ineffectiveness of repeated passage in piglets as a feedback practice to control PEDV outbreaks on farms.

Therefore, the present study spotlights the idea that increasing passages of PEDV leads to changes in the S and ORF3 genes, affecting the virus's ability to infect cells and results in decreased virulence. Furthermore, our study confirms that in the in vivo passage model, virulence of PEDV is markedly reduced as passage numbers increase. This explains the field observations of decreased virulence with time. This suggests that over time, PEDV may evolve towards an avirulent state in swine with natural passage through hosts. The transition of PEDV to an avirulent state in the host warrants further exploration. In addition to understanding of PEDV, this exploration also sheds light on the evolution and co-existence of novel coronaviruses with the host through a similar mechanism.

## Materials and methods

All animal procedures were conducted following the recommendations in the Guide for the Care and Use of Laboratory Animals of the National Research Council of Thailand according to protocols reviewed and approved Faculty of Veterinary Science, Mahidol University-Institute Animal Care and Use Committee (FVS-MU-IACUC; animal use license number U1-01281-2558). All methods were performed in accordance with the relevant guidelines and regulations. The study is reported in accordance with the ARRIVE guidelines (https://arriveguidelines.org).

### Cells and virus

Vero C1008 cells were obtained from ATCC (ATCC^®^ CRL-1586™). Vero C1008 cells were cultured in Dulbecco’s Modified Eagle Medium (DMEM; Gibco, New York, USA) supplemented with antibiotics (100 units/mL of penicillin, 100 μg/mL of streptomycin, and 0.25 μg/mL of Fungizone^®^ (Life Technologies, New York, USA), GlutaMAX™-I (Life Technologies, New York, USA) and 10% heat inactivated fetal bovine serum (Gibco, New York, USA) in T75 flask (Corning^®^, New York, USA) and maintained at 37 °C in a humidified 5% CO_2_ incubator.

The PEDV isolate used as a parental strain in the study was PED-JPFP0-PJ. The PED-JPFP0-PJ strain was isolated from intestinal samples of three-day-old piglets displaying clinical symptoms indicative of PEDV, including vomiting, watery diarrhea and high mortality. Intestinal samples were collected during an acute PEDV outbreak from a swine farm located in the western region of Thailand, The PED-JPFP0-PJ isolate is classified in genotype G2, based on S gene. The datasets of complete S and ORF3 gene sequences of PED-JPFP0-PJ isolate and its progeny viruses generated and/or analyzed during the current study are available in the GenBank repository, [PP176560-PP176625]. PED-JPFP0-PJ was isolated and propagated in Vero C1008 cells. In brief, to isolate the virus, 1 g of minced intestine effected by PEDV infection were suspended in 10 mL of 1X PBS (1X phosphate-buffered saline; 0.1 M, pH 7.2). Following a slow mixing, the suspension was then centrifuged at 4800×*g* for 10 min. Supernatant was filtered through 0.45 and 0.22 µm filters, respectively, and stored at − 80 °C until use.

### Serial passages in vitro for PEDV

Following the isolation, the parental isolate, PED-JPFP0-PJ, was serially in vitro passaged for 5 consecutive times in Vero C1008 cells. Progeny viruses from the 1st to 5th passages were recognized as PED-JPFP1-PJ, PED-JPFP2-PJ, PED-JPFP3-PJ, PED-JPFP4-PJ, and PED-JPFP5-PJ, respectively.

To perform an in vitro serial passage, after Vero C1008 cells reaching approximately 80% confluency, the cells were washed twice with 1X PBS, and then incubated with 1 mL of PED-JPFP0-PJ for 60 min at 37 °C, 5% CO_2_, followed by adding 20 mL of maintenance media (DMEM supplemented with antibiotics, 100 units/mL of penicillin, 100 μg/mL of streptomycin, and 0.25 μg/mL of Fungizone^®^) (Life Technologies, New York, USA) and 8 µg/mL trypsin/EDTA (Gibco, New York, USA). The PEDV inoculated Vero cells were observed daily for the development of cytopathic effect (CPE) including syncytia formation, cell fusion and partially detached from surface monolayer. After CPE were observed, the inoculated cells were frozen at −80 °C. Freeze-thaw cycles were performed twice, and the virus was collected by centrifuge at 2500 rpm for 30 min. The virus was stored at −80 °C for further passages.

In each passage, virus titer and sequencing data of S and ORF3 genes were determined. Virus titration was performed in accordance with the method previously described by Reed-Muench^39^. In brief, each passage of PEDV was tenfold serially diluted in DMEM supplemented with antibiotics 100 units/mL of penicillin, 100 μg/mL of streptomycin, and 0.25 μg/mL of Fungizone^®^ (Life Technologies, New York, USA) and 8 µg/mL trypsin/EDTA (Gibco, New York, USA). 100 µL of each dilution were inoculated into 80% confluency, Vero C1008 cells sub-cultured in a 96-well microplate (Corning^®^, New York, USA). The inoculated cells were incubated for 7 consecutive days at 37 °C, 5% CO_2_, and CPE was observed daily. Complete S and ORF3 gene sequences were genetically characterized using specific primers (Supplementary Table S1) in accordance with protocols described below.

### Experimental design

Thirty-six 3-day-old piglets were procured from a herd with no history of either PEDV or PDCoV outbreak. The negative status of incoming piglets against PEDV, PDCoV, TGEV, swine acute diarrhea syndrome coronavirus (SADS-CoV) and porcine rotavirus (groups A, B and C) were confirmed using PCR methods. In brief, total viral RNA was extracted from rectal swabs using Nucleospin^®^ viral RNA isolation kit (Macherey–Nagel Inc., Duren, Germany) and conversed to cDNA using M-MuLV Reverse Transcriptase (Biolabs Inc., Ipswich, Massachusetts, USA). PCR was performed using 5 × HOT FIREPol^®^ Blend Mastermix with 10 mM MgCl_2_ (Solis Biodyne, Tartu, Estonia). Four specific primers were used to detect PEDV M gene, PDCoV N gene, TGEV S gene and SADS-CoV RdRp gene^40^. For porcine rotavirus, specific primers against VP4, NSP2, and VP6 genes were used to detected porcine rotavirus group A, B, and C, respectively^41^.

Thirty-six piglets were randomly allocated into 3 batches of 12 piglets each (Supplementary Fig. S1). The first, second and third batches of piglets were called F1, F2 and F3, respectively. In the first batch (F1), 4 groups designated as P0F1 (n = 3), P2F1 (n = 3), P5F1 (n = 3) and controlF1 (n = 3). The P0F1, P2F1 and P5F1 were inoculated orally with 2 mL of PED-JPFP0-PJ, PED-JPFP2-PJ, and PED-JPFP5-PJ, respectively, at a titer of 3 log_10_ TCID_50_/mL. Piglets in the control group were inoculated orally with 2 mL of DMEM. At 24 h-post-inoculation (HPI), all piglets were euthanized by an intravenous injection of sodium pentobarbital overdose. Necropsy was performed immediately after euthanasia, intestinal samples were collected and designated as P0F1, P2F1, P5F1 and controlF1. These intestinal materials were subjected for further analyses including quantification of PEDV genome, macroscopic examination, villous height and crypt-depth (VH:CD) ratio, immunohistochemistry, and genetic characterization of S and ORF3 genes, and the oral inoculation for the second batch of piglets, designated F2.

Three intestinal samples from each group were pooled and homogenized before inoculation to the next batch. In the second batch of piglets, 1 g of homogenized intestinal samples from P0F1, P2F1 and P5F1 groups were suspended in 10 mL of 1X PBS. The suspensions were then filtered through 0.45 and 0.22 µm filters. Quantitative real-time polymerase chain reaction (qPCR) was performed to quantify genomic copy number of inoculum material according the protocol already described^42^. The inoculum contained 3 log_10_ genomic copies/mL. The F2 piglets were orally inoculated with 2 mL of intestine suspension of P0F1, P2F1, and P5F1. At 24 HPI, all F2 pigs were euthanized. Intestines were collected and designated as P0F2, P2F2, and P5F2. These intestinal materials were subjected for further analyses and the oral inoculation for the third batch of piglets, designated F3. In the third batch, the piglets designated as P0F3, P2F3 and P5F3 were orally inoculated with 2 mL of intestinal samples from P0F2, P2F2, and P5F2, respectively.

Small intestinal samples of F2 and F3 piglets were collected and processed in accordance with samples of F1 piglets. The negative control group in each batch was orally inoculated with 2 mL of previous negative intestine suspension batch. To determine virulence of the virus between each in vivo passage, quantification of PEDV genome was performed in all intestine samples using qPCR as described^43^.

### Pathological examination

To perform macroscopic examination, 5 parts of small intestine including duodenum, proximal jejunum, middle jejunum, distal jejunum, and ileum were collected as both fresh and 10% formalin-fixed specimens. Gross lesions including thin transparent wall of intestines, and yellowish content fluid in intestinal lumen were determined. The formalin-fixed specimens were subjected for further analyses including microscopic examination by villous height and crypt-depth (VH:CD) ratio, and antigen detection by immunohistochemistry (IHC).

For microscopic examination, VH:CD ratio was determined in 5 parts of small intestine including duodenum, proximal jejunum, middle jejunum, distal jejunum, and ileum. In brief, 10 representative views of each section were randomly selected to measure villous height and crypt depth using a computerized image system (Olympus IX73 camera, Tokyo, Japan). VH:CD ratio was calculated based on the quotient outcome of the average villi height divided by the average crypt depth using cellSens Dimension 1.16 digital imaging software.

### Immunohistochemistry (IHC)

Briefly, formalin-fixed, paraffin-embedded (FFPE) specimens were prepared and placed on positive charge glass slide (Thermo Fisher Scientific, Waltham, MA, USA). The paraffin sections were incubated at 60 °C for paraffin melting, followed by deparaffinization in xylene.

Immunohistochemistry was performed using mouse monoclonal anti-Nucleocapsid (N) protein (clone SD6-29) of PEDV (Medgene Labs, Brooking, SD). Tissues were processed and placed on Superfrost Plus slides (Thermo Fisher Scientific, Waltham, MA, USA). Sections were deparaffinized, rehydrated using an alcohol gradient and air-dried. All slides were treated with proteinase K (Thermo Fisher Scientific, Waltham, MA, USA) in PBS for 30 min. Endogenous alkaline phosphatase was quenched with 0.3% hydrogen peroxide for 5 min. All slides were then incubated with BSA for 30 min. The slides were separately incubated with monoclonal antibodies overnight at 4 °C in a humidified chamber. After washing, PEDV-N antigen was visualized by binding with secondary antibody conjugated with horseradish peroxidase conjugated (HRP)-labeled polymer followed by immersion in peroxidase (Dako REAL™ Envision™/HRP, rabbit/mouse (ENV), Dako, Copenhagen, Denmark).

Intestinal tissues from pigs in the unchallenged group served as negative controls. To obtain quantitative data, slides were analyzed with the NIH Image J 1.50i Program (http://rsb.info.nih.gov/ij). In each slide, 10 fields were randomly selected, and the number of positive cells per unit area (0.95 mm^2^) was determined as previously described^15^. The mean values were calculated. IHC score was determined by semi-quantitatively scored assay under inverted microscope that performed and interpreted by using previously described method^42,44^.

Briefly, antigen retrieval was observed in villous and crypt enterocytes positive staining based on the following criteria; 0 = no staining; 1 = 1–10% of positive staining; 2 = 10–25% of villous enterocytes positive signal; 3 = 25–50% of positive staining in section; and 4 = 50–100% of positive signal in enterocyte.

### Sequencing and in silico analysis

#### Reverse transcription and polymerase chain reaction

The propagated PED-JPFP0-PJ strain in Vero C1008 cells was confirmed, total RNA was extracted from supernatant using Nucleospin^®^ viral RNA isolation kit (Macherey–Nagel Inc., Duren, Germany) in accordance with manufacturer’s instructions. Viral cDNA was generated using M-MuLV Reverse Transcriptase (Biolabs Inc., Ipswich, MA, USA). The cDNA was then used to amplify the complete S gene by polymerase chain reaction (PCR) using 5× HOT FIREPol^®^ Blend Mastermix with 10 mM MgCl_2_ (Solis Biodyne, Tartu, Estonia) together with specific primers (Supplementary Table S1). To clarify the isolate was not contaminated with SADS-CoV. The cDNA was amplified RdRp gene of SADS-CoV using specific primer^40^. PEDV positive results were sequenced at First BASE Laboratory (Selangor, Malaysia).

Parental PED-JPFP0-PJ strain, five passages in cell culture, and all intestinal samples were used to extract total viral RNA and reverse to cDNA as described above. To amplify complete S and ORF3 genes, the cDNA was used to perform PCR amplification using 5× HOT FIREPol^®^ Blend Mastermix with 10 mM MgCl_2_ (Solis Biodyne, Tartu, Estonia) together with 6 pairs of primers for S gene and 1 pair of primer for ORF3 gene (Supplementary Table S1). Positive bands were visualized on agarose gel electrophoresis. The positive bands were purified using the Nucleospin Gel and PCR Clean-up kit (Macherey–Nagel Inc., Bethlehem, PA, USA) and subjected for sequencing. Sequencing was performed at First BASE Laboratory (Selangor, Malaysia). Nucleotide and deduced aa were analyzed by CLUSTALW program implemented in BioEdit Sequence Alignment Editor^45^.

### Sequence variation analysis

To analyze variation of nt and aa sequences based on complete S and ORF3 genes. The sequences were aligned using CLUSTALW program implemented in BioEdit Sequence Alignment Editor^45^. The percentage of similarities between the isolates were computed at both nt and aa levels. Mutation analysis of nucleotides (substitution per nucleotide per cell infection; s/n/c) were demonstrated using BEAST packages (v1.10.4)^46^ using Bayesian skyline prior model and uncorrelated lognormal relaxed molecular clock with logged every 10,000 states at least a thousand million state to qualify ESS values > 200, 10% discarded was applied for the states using in both analysis, GTR + G was chosen as the best-fit model using MEGA-X for both analyses.

### Hydrophilicity and antigenicity analysis

Amino acid sequences of S and ORF3 sequences, including PED-JPFP0-PJ and its lineage sequences, were used in hydrophilicity and antigenicity analyses. CLUSTALW program implemented in BioEdit Sequence Alignment Editor was used to align the protein sequences^45^. To identify hydrophilicity and antigenicity based on the aa sequences. Hopp-Woods hydrophilicity plot and Kolaskar & Tongaonkar antigenicity were created using Protscale Expasy (http://web.expasy.org/protscale) and IEDB Analysis Resource (http://tools.immuneepitope.org/main), respectively.

### Secondary structure prediction

To examine surface proteins. The first (F1) and the last (F3) in vivo passages of each in vitro serial passage (P0F1, P0F3, P2F1, P2F3, P5F1 and P5F3) were subjected for the prediction of the secondary structure, compared to that of the parent isolate, PED-JPFP0-PJ. The secondary structure was predicted using SWISS-MODEL Workspace^47^. Figures were ornamented using SWISS-MODEL and Swiss Pdb Viewer programs^48^.

### Statistical analyses

All data, displayed as mean ± standard deviation, of PEDV titers, genomic copy numbers, VH:CD ratios, and IHC scores were analyzed using GraphPad 213 Prism 9 (GraphPad Software Inc., La Jolla, CA, USA). Analysis of variance (ANOVA) was performed to compare the means across each passage group. The PEDV titers were compared between parent isolate, PED-JPFP0-PJ, and its 1st to 5th serial passages in vitro. PEDV genomic copy numbers between batch serial passage in vivo was determined and compared between batch. VH:CD ratios and IHC scores between parts of intestine were calculated and compared. Significant differences between passage groups at *p* value ≤ 0.05 were subjected for further analyses using Tukey's multiple comparison.

### Supplementary Information

Supplementary Information.

## Data Availability

The datasets of complete S and ORF3 gene sequences of PED-JPFP0-PJ isolate and its progeny viruses generated and/or analyzed during the current study are available in the GenBank repository, [PP176560-PP176625]. The PEDV S glycoproteins have been deposited in the Worldwide Protein Data Bank with the accession code 7w6m. The other datasets generated during and/or analysed during the current study are available from the corresponding author on reasonable request.
