# Supplementary material for: Evolution and virulence of porcine epidemic diarrhea virus following in vitro and in vivo propagation
Source: Sci Rep. 2024 May 29;14:12279. doi: 10.1038/s41598-024-62875-6 (PMC11137156; doi:10.1038/s41598-024-62875-6)
Supplement: Supplementary file 1 — Supplementary Information. [file 41598_2024_62875_MOESM1_ESM.docx]

**Supplementary information**


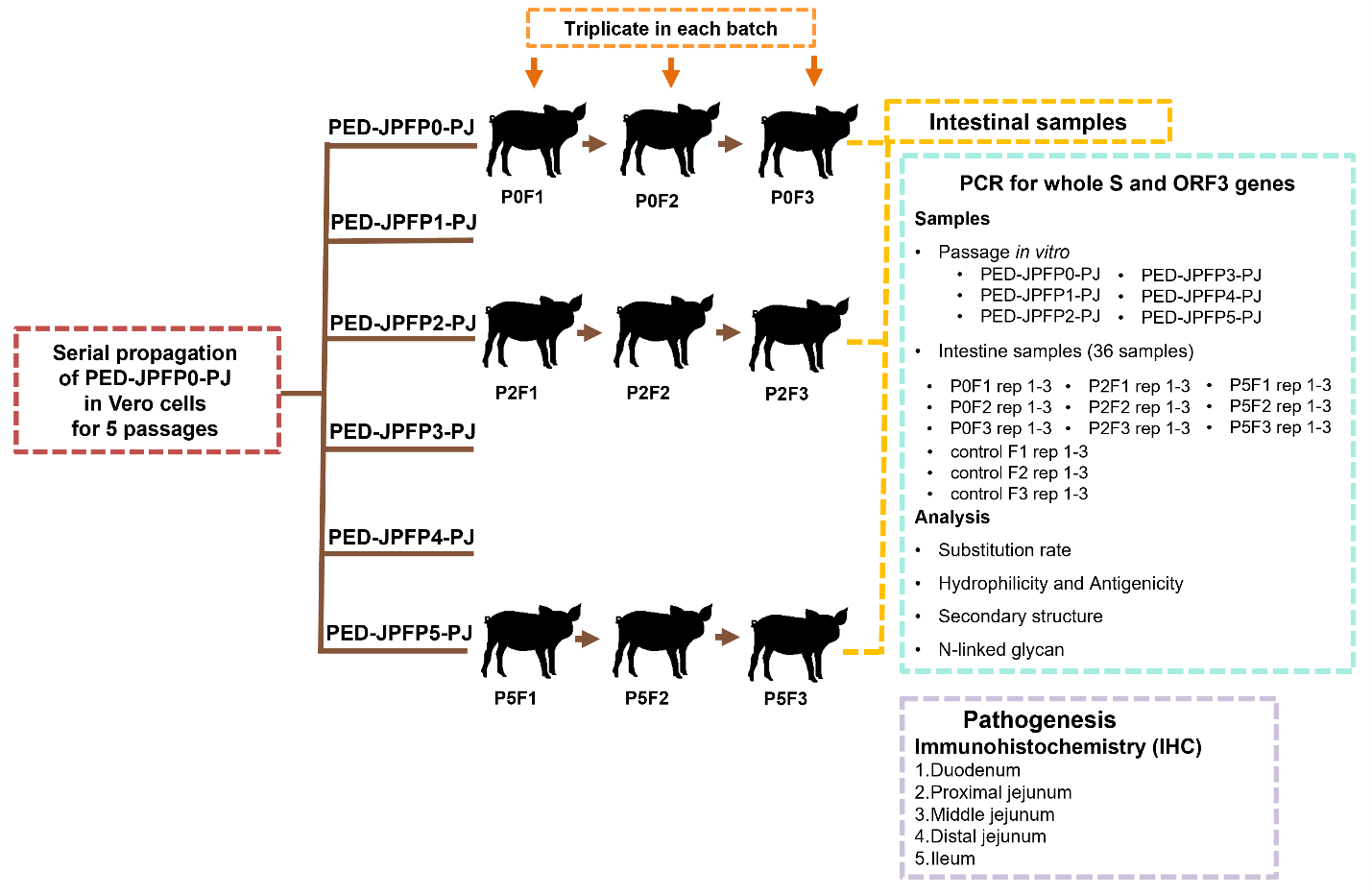


**Fig S1.** Experimental Design. This diagram depicts two parts of the study, including *in vitro* and *in vivo* phases. PED-JPFP0-PJ, PED-JPFP2-PJ, and PED-JPFP5-PJ obtained from the *in vitro* phase were selected to inoculate three-day-old piglets, designated as F1. Piglets in batches F2 and F3 were inoculated with 2 mL of intestinal suspension at a titer of 3 log_10_ TCID_50_/mL from a previous *in vivo* passage. Various analyses including substitution rate, hydrophilicity, antigenicity, secondary structure, and N-linked glycan analysis were performed. Villous height and crypt-depth (VH:CD) ratio, as well as immunohistochemistry (IHC), were conducted to examine the virulence and target of PEDV infection.


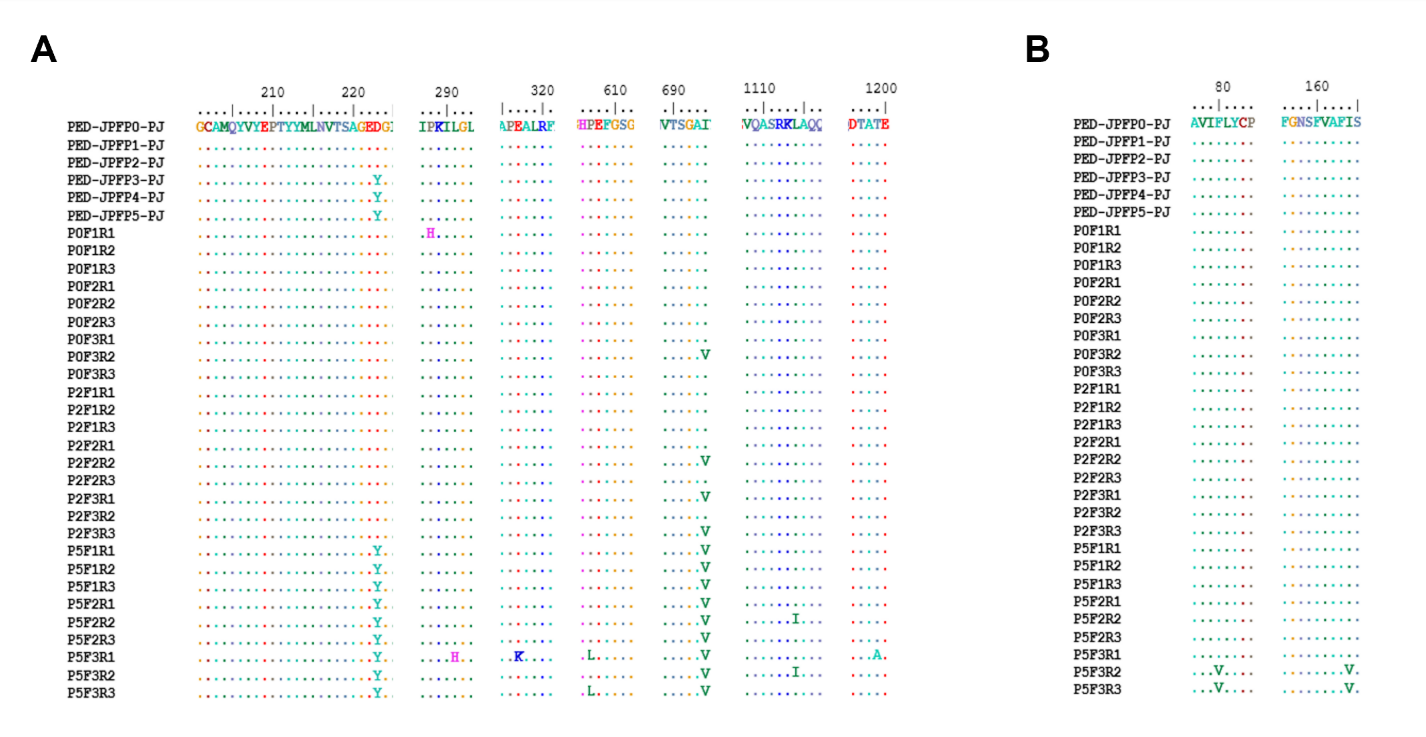


**Fig S2.** Alignment of amino acid for the whole S protein (A) and ORF3 protein (B) of PED-JPFP0-PJ and its variants, both *in vitro* and *in vivo*. Each box represents aa substitutions in its variants compared to PED-JPFP0-PJ.

**Table S1.** **Whole spike (S) and ORF3 genes primers.**

| Target genes | Primers |
| --- | --- |
| Whole S gene | S_F1: 5´-TGCTAGTGCGTAATAATGACGCCAT-3´ |
|  | S_R1: 5´-CATCCTCACCAGCACTAGTAACATT-3´ |
|  | S_F2: 5´-GATTGGTCCCGTGTTGCGACA-3´ |
|  | S_R2: 5´-CTGAGAACACTTGAGTTGGCTAACA-3´ |
|  | S_F3: 5´-GTCAGGGAAATTGTCATCACCAAGT-3´ |
|  | S_R3: 5´-CAGAATAAACAGCACCACTAGTGAC-3´ |
|  | S_F4: 5´-ACGGACGTTTCTTTTATGACTCTGG-3´ |
|  | S_R4: 5´-GAGCGACCATTAGAACAGCGC-3´ |
|  | S_F5: 5´-TGCAAGTGGCAGGGTGGTACA-3´ |
|  | S_R5: 5´-GCCAGGCTCACGTAGAGTCAA-3´ |
|  | S_F6: 5´-ATGTTATTGCCATCGCTGGCTTATG-3´ |
|  | S_R6: 5´-ACAAAGCCTGCCAATAAGTGTGCA-3´ |
| Whole ORF3 gene | ORF3_F: 5´-CCTAGACTTCAACCTTACGA-3´ |
|  | ORF3_R: 5´-CAGGAAAAAGAGTACGAAAA-3´ |

**Table S2.** **Mean ± SEM (standard error mean) values for** **substitution rate base on full-length spike gene and ORF3 gene.**

|  | Genes | |
| --- | --- | --- |
|  | Spike | ORF3 |
| PED-JPFP0-PJ to PED-JPFP5-PJ | 4.01 x 10^-4^ (**±**4.70 x 10^-7^) | 8.17 x 10^-5^ (**±**5.35 x 10^-7^) |
| P0F1 to P0F3 | 3.47 x 10^-4^ (**±**1.14 x 10^-6^) | 1.29 x 10^-4^ (**±**8.66 x 10^-7^) |
| P2F1 to P2F3 | 1.67 x 10^-4^ (**±**1.17 x 10^-6^) | 1.28 x 10^-4^ (**±**8.61 x 10^-7^) |
| P5F1 to P5F3 | 1.04 x 10^-3^ (**±**1.17 x 10^-6^) | 5.61 x 10^-4^ (**±**1.37 x 10^-5^) |
| PED-JPFP0-PJ to P5F3 | 4.16 x 10^-4^ (**±**4.43 x 10^-7^) | 5.37 x 10^-4^ (**±**1.85 x 10^-5^) |

***Mean (±SEM)**
